# Comparative physiological, metabolomic and transcriptomic analyses reveal the mechanisms of differences in pear fruit quality between distinct training systems

**DOI:** 10.1186/s12870-023-04716-8

**Published:** 2024-01-04

**Authors:** Zheng Liu, Xie-Yu Li, Li Yang, Yin-Sheng Cheng, Xian-Shuang Nie, Tao Wu

**Affiliations:** Hubei Key Laboratory of Germplasm Innovation and Utilization of Fruit Trees, Research Institute of Fruit and Tea, Hubei Academy of Agricultural Sciences, Wuhan, Hubei 430064 China

**Keywords:** Pear fruits, Training systems, Canopy environment, Metabolome, Transcriptome, Phenylpropanoid biosynthesis pathway

## Abstract

**Background:**

Canopy architecture is critical in determining the fruit-zone microclimate and, ultimately, in determining an orchard’s success in terms of the quality and quantity of the fruit produced. However, few studies have addressed how the canopy environment leads to metabolomic and transcriptomic alterations in fruits. Designing strategies for improving the quality of pear nutritional components relies on uncovering the related regulatory mechanisms.

**Results:**

We performed an in-depth investigation of the impact of canopy architecture from physiological, metabolomic and transcriptomic perspectives by comparing pear fruits grown in a traditional freestanding system (SP) or a flat-type trellis system (DP). Physiological studies revealed relatively greater fruit sizes, soluble solid contents and titratable acidities in pear fruits from DP systems with open canopies. Nontargeted metabolite profiling was used to characterize fruits at the initial ripening stage. Significant differences in fruit metabolites, including carbohydrates, nucleic acids, alkaloids, glycerophospholipids, sterol lipids, and prenol lipids, were observed between the two groups. Transcriptomic analysis indicated that a series of organic substance catabolic processes (e.g., the glycerol-3-phosphate catabolic process, pectin catabolic process and glucan catabolic process) were overrepresented in fruits of the DP system. Moreover, integrative analysis of the metabolome and transcriptome at the pathway level showed that DP pear fruits may respond to the canopy microenvironment by upregulating phenylpropanoid biosynthesis pathway genes such as *PpPOD*. Transient assays revealed that the contents of malic acid and citric acid were lower in the pear flesh of *PpPOD* RNAi plants, which was associated with regulating the expression of organic acid metabolism-related genes.

**Conclusions:**

Our results provide fundamental evidence that at the physiological and molecular levels, open-canopy architecture contributes to improving pear fruit quality and is correlated with increased levels of carbohydrates and lipid-like molecules. This study may lead to the development of rational culture practices for enhancing the nutritional traits of pear fruits.

**Supplementary Information:**

The online version contains supplementary material available at 10.1186/s12870-023-04716-8.

## Background

Training and pruning are important horticultural techniques for managing the architecture of canopies; these techniques influence plant density, light interception and distribution, which in turn impact yield and fruit quality [1, 2]. Microclimate gradients inside tree canopies may alter leaf phenology, photosynthetic carbon assimilation rates and fruit quality [3–6]. Therefore, to produce high-quality fruits, the goal of suitable tree architecture should be to optimize the canopy microenvironment. Understanding the molecular mechanisms underlying canopy microenvironmental responses in fruits may contribute to the development of rational approaches for maximizing orchard quality potential.

It is well established that the microenvironment around individual fruits can influence metabolite compositions and gene expression profiles and consequently impact their external and internal quality [7, 8]. The fruit-zone microenvironment has been implicated in affecting the profiles of primary (such as amino acids and organic acids) and secondary metabolites (such as phenylpropanoids, flavonoids, flavonols, and triterpenoids) [9, 10]. Both primary and secondary metabolites are valuable components of nutritional and organoleptic characteristics of fruit [11–13] and are closely associated with transcriptional regulatory mechanisms [14]. For instance, a microenvironment produced by bagging resulted in reduced carotenoid levels in peach fruits, which may be due to the negative regulation of the expression of carotenoid structural genes by *PpPIF3* [15]. However, the effects of tree architecture on fruit quality, especially the molecular mechanisms of metabolomics and transcriptomic alterations, still need to be better understood.

Pear is one of the most important fruit crops worldwide. Canopy architecture is critical for the microenvironment of leaves and fruit development and has multiple effects on photosynthetic efficiency and fruit quality [16]. In our previous study, we found that the flat-type trellis system (double primary branches above the row, DP) presented higher net photosynthetic rates, and photosynthesis, carbohydrate catabolic processes and fatty acid metabolic processes were overrepresented in the leaves of DP systems with open-canopy characteristics [17]. *PpPRR5* was shown to be associated with negatively regulating photosynthetic performance under distinct training systems [18]. However, the mechanism by which canopy architecture affects pear fruit quality is not fully understood. The application of large-scale molecular methods (“high-throughput omics”) is a particularly important approach for advancing our understanding of the complex biological processes associated with fruit maturation and quality development [19]. Previous studies have utilized metabolomic and/or transcriptomic methods to investigate the impact of the canopy environment on fruit quality in peach or grape plants [2, 20], but limited information is available for pear plants. In the present study, comparative physiological analysis was performed to investigate the phenotypic differences in pear fruits grown in distinct training systems. Moreover, we used a metabolomics approach to characterize changes in various pear fruit metabolites under distinct training systems. Through comparative transcriptome analysis, dozens of key biological processes and regulatory pathways that are influenced by canopy environments were identified. This study offers novel insights into how fruit crops systematically react to heterogeneous microclimate conditions and therefore aims to improve canopy environments in pear orchards.

## Methods

### Plant materials

Ten-year-old ‘Wonhwang’ (*Pyrus pyrifolia* Nakai cv. Wonhwang) pear fruits were collected from the research orchard (30.292°N, 114.143°E) of the Research Institute of Fruit and Tea. All the trees were spaced 4 m apart, and the rows were 3 m wide. The trees were trained on a traditional freestanding system (delayed-open central leader, SP) or a DP system as described previously [17, 21]. The trees in the SP system had a classically central, vertical leader and 3–5 primary branches. In this system, light penetration to the interior part of the canopy (IN) was obstructed. In the DP system, the two permanent primary branches were trained upwards into a Y shape along the row. The Y junction was 1.5 m above the ground, and the angle was 45°. A few side branches, which were temporarily retained as fruiting branches on the primary branches for one to two years, were drawn naturally on a horizontal wire trellis approximately 1.8 m high. The fruit load intensity of the trees in the orchard was determined at the beginning of April. The experiment was laid out in accordance with a randomized complete block design. Uniform heavily producing trees within each block were randomly selected and represented biological replicates per the training system. Each tested tree was divided into two fruit locations, i.e., IN and the exterior part of the canopy (EX). The trees were managed according to standard horticultural practices and insect control. Fruits at different developmental stages were collected at 60, 90 and 120 days after flowering (DAF).

‘Yulv’ (*P. pyrifolia* Nakai cv. Yulv) fruits were selected at 60 DAF and subjected to the *PpPOD* virus-induced gene silencing (VIGS) assay. All the fruits were frozen in liquid nitrogen immediately and stored at -80 °C until use.

### Determination of fruit size, total soluble solids and titratable acidity

To determine the average size of the fruits, two linear dimensions, fruit length and equatorial fruit diameter, were measured by using a digital calliper with a sensitivity of 0.01 mm according to Zhang et al. [22]. The soluble solid content (SSC) and titratable acidity (TA) were measured from the pressed juice of each fruit sample using a pocket Brix-acidity meter (PAL-BX/ACID 1, Atago Co., Ltd., Japan) according to the manufacturer’s instructions [23]. SSC and TA are expressed as Brix percentages and acidity, respectively. For greater precision, three biological replicates for each sample and three technical replicates (three fruits) of each biological replicate were analysed. The fruits collected from three individual trees were defined as three biological replicates and were used to calculate the standard deviation. All the statistical analyses were performed using IBM SPSS Statistics 19 software. Duncan’s multiple range test was used to determine significant differences between different samples (*P* < 0.01).

### Metabolite extraction and liquid chromatography–mass spectrometry (LC–MS) analysis

Samples of pear fruits at the initial ripening stage (120 DAF) were selected for metabolomic analysis. Metabolites were extracted from lyophilized fruit samples (six biological replicates) using the method of Xu et al. [24] with minor modifications. The samples were ground to a fine powder in liquid nitrogen and then subjected to metabolite extraction in methanol/water (4:1, v/v) solution. Subsequently, the mixture was allowed to settle at -20 °C for 10 min, crushed at 50 Hz for 6 min, and ultrasonicated at 40 kHz for 30 min at 5 °C. Then, the extracts were incubated at -20 °C for 30 min to precipitate the proteins. After centrifugation at 13,000 × g for 15 min, the supernatant was carefully transferred to a sample vial for LC–MS/MS analysis. To monitor the stability and repeatability of the instrumental analysis, quality control samples were established by pooling equal volumes of each sample, and the samples were tested in the same manner as the analytic samples.

Next, following the method described by Wu et al. [25], untargeted metabolite profiling was carried out on a Thermo UHPLC system equipped with an ACQUITY BEH C18 column (100 mm × 2.1 mm i.d., 1.7 μm; Waters Corporation, Milford, USA). The mobile phase was composed of solvent A (0.1% formic acid in water) and solvent B (0.1% formic acid in acetonitrile: isopropanol; 1:1, v/v). The flow rate of the mobile phases was 0.4 mL/min. The MS data were acquired in positive and negative ion modes (full scan mode from 70 − 1,050 m/z). The key parameters of the operating conditions were as follows: aus gas heater temperature of 400 °C, sheath gas flow rate of 40 psi, aus gas flow rate of 30 psi, and ion-spray voltages floating at 3500 V in positive mode and − 2800 V in negative mode.

Metabolomic data processing was performed as described by Wu et al. [25]. Orthogonal partial least squares-discriminant analysis (OPLS-DA) was performed to compare the metabolic differences between the experimental groups. The quality of the models was assessed by the cumulative modelled variation in the X and Y matrix (*R*^*2*^*X* and *R*^*2*^*Y*) and the cross-validated predictive ability *Q*^*2*^ (cum) values. Differentially regulated metabolites (DRMs) were determined based on the combination of a statistically significant threshold of variable importance in the projection (VIP > 1.0) and Student’s *t* test (*P* < 0.05). The differential pathway enrichment analysis was based on the Kyoto Encyclopedia of Genes and Genomes (KEGG) database (https://www.kegg.jp/kegg/kegg1.html) [26]. Pathways with *p* < 0.05 were considered significantly different.

### RNA isolation and RNA-seq

Total RNA was extracted from the frozen fruits using an RNAprep Pure Plant Kit (Polysaccharides & Polyphenolics-rich) (Tiangen, China) according to the manufacturer’s protocol. A NanoPhotometer™ spectrophotometer (IMPLEN, Germany) and an Agilent 2100 Bioanalyzer (Agilent Technologies, USA) were used to measure the concentration and quality of the RNA, respectively. For each developmental stage of each training system, the RNA from the IN and EX fruits were equimolarly pooled and used as a single sample for transcriptome sequencing. Fruits collected from three trees were used as three biological replicates.

cDNA library construction and sequencing were performed by Majorbio Biopharm Technology Co., Ltd. (Shanghai, China). All RNA-seq data were submitted to the NCBI Sequence Read Archive and assigned the accession number PRJNA967128. Clean reads were obtained by removing adaptor sequences and ambiguous nucleotides using SeqPrep (https://github.com/jstjohn/SeqPrep) and Sickle (https://github.com/najoshi/sickle). The high-quality clean data were mapped to the reference genome of pear (http://peargenome.njau.edu.cn/) with Bowtie2 software (https://sourceforge.net/projects/bowtie-bio/files/bowtie2/2.3.5.1/) [27]. Principal component analysis (PCA) was performed to obtain an overview of the sample distribution [28]. The gene expression levels were calculated and normalized via fragments per kilobase per million reads (FPKM) values [29]. Differentially expressed genes (DEGs) were defined by a |log_2_(fold change)| ≥ 1 and adjusted *p* < 0.05. The overlapping DEGs were analysed using VennDiagram [30]. To assess the distribution of DEG functions, Gene Ontology (GO) enrichment analysis was performed using Goatools software and Fisher’s exact test (http://www.geneontology.org/) [31]. The GO terms with *p* < 0.05 were defined as significantly enriched. Pathway enrichment analysis was implemented by using the KEGG database (https://www.kegg.jp/kegg/kegg1.html) [26]. Enriched KEGG pathways were analysed using default parameters (*p* < 0.05) and then plotted using Microsoft Excel software.

### Quantitative real-time PCR (qRT–PCR) analysis

cDNA was synthesized from total RNA using a RevertAid™ First Strand cDNA Synthesis Kit (Fermentas, USA) following the manufacturer’s instructions [21]. Primer Premier 5.0 software was used to design qRT–PCR primer pairs for the selected genes (Additional file 1). Each run was completed with a melting curve for the tested genes to confirm the specificity of the amplification. The relative gene expression was measured using the 2^−ΔΔ*C*T^ method with the reference genes *PpSKD1* and *PpYLS8* according to our previous studies [21]. Standard errors were calculated based on three biological replicates.

### RNAi transient expression assay of pear fruit and measurement of organic acid contents

The 505 bp fragments of the C-terminus of PbPOD were PCR-amplified (Additional file 1) and inserted into the multiple cloning site (BamHI-Xhol) of pTRV2 to construct the pTRV2-*PbPOD* vector (*PbPOD*-TRV). The VIGS assay was performed as previously described [32, 33]. The *Agrobacterium* strain GV3101 containing pTRV1 and pTRV2 was used independently, and their derivatives were used for transient expression experiments. The negative controls were infiltrated with *Agrobacterium* containing the pTRV2 empty vector.

The organic acid contents in the fruit were determined as described by Li et al. [34] with minor modifications. Approximately 0.5 g of ground flesh was mixed with 1.0 ml of metaphosphoric acid (0.2 M) and then exposed to ultrasonic irradiation for 30 min to promote extraction. The extraction mixture was centrifuged at 11,000 × g and 4 °C for 10 min, after which the supernatant was filtered through a 0.22 μm microporous membrane and analysed via HPLC. Chromatographic separation was performed on an Athena C18-WP column (CNW, 5 μm, 250 × 4.6 mm) at a flow rate of 1.0 mL/min at 30 °C. The mobile phase consisted of 0.01 M potassium dihydrogen phosphate buffer (pH = 2.65). Six biological replicates were performed. The data were subjected to analysis of variance (ANOVA), and mean comparisons were conducted by Student’s t test with a significance level of *p* < 0.05.

## Results

### Physiological differences in pear fruits under different canopy architectures

**Fig. 1:**
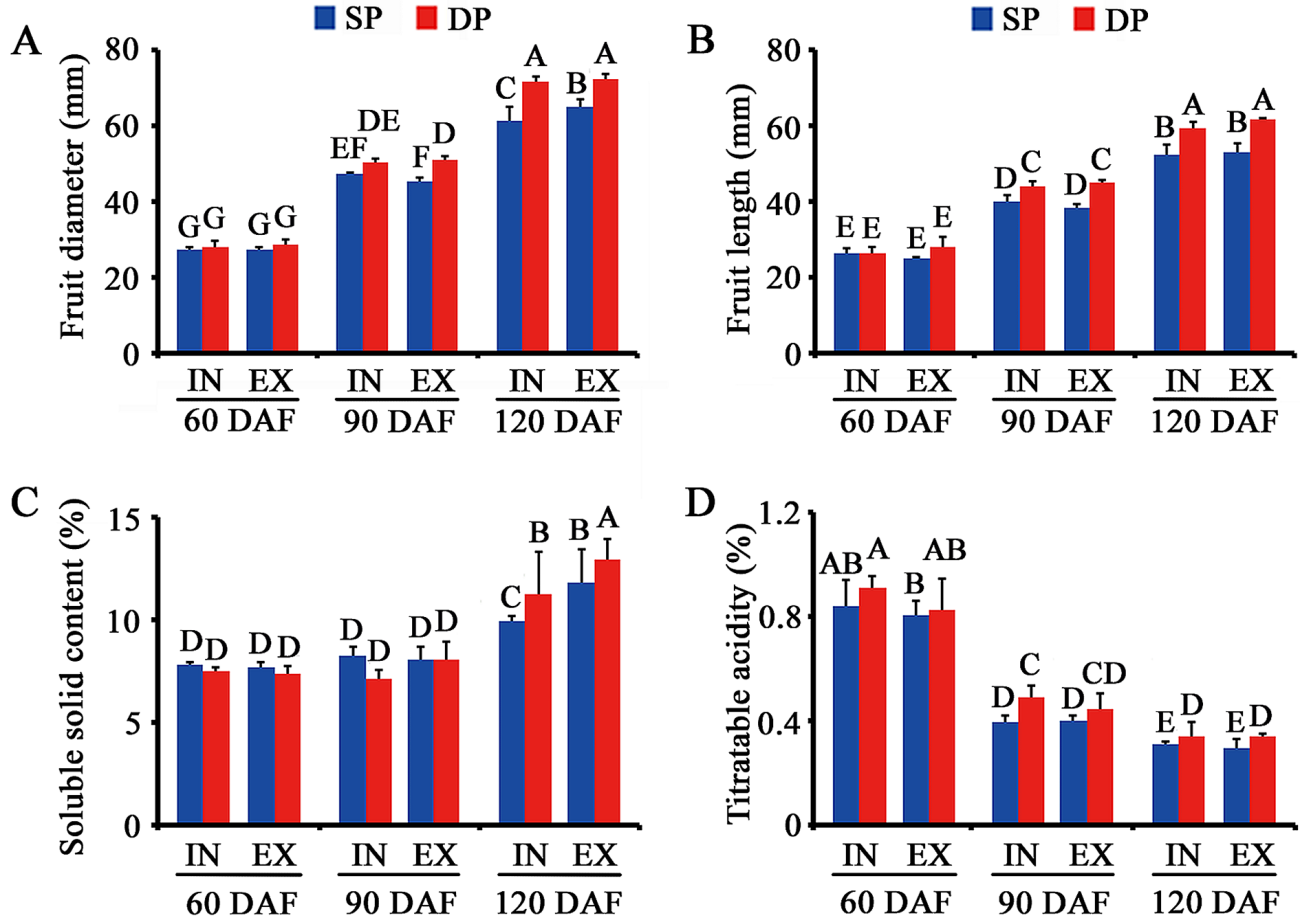
Effects of distinct training systems on fruit diameter (**A**), fruit length (**B**), soluble solid content (**C**) and titratable acidity (**D**) during pear fruit development. Samples from the SP (traditional freestanding system) and DP (flat-type trellis system) were collected at 60 DAF (days after flowering), 90 DAF and 120 DAF. IN/EX: Fruits in the interior/exterior part of the canopy. The data are presented as the mean ± SD (n = three biological replicates). The capital letters above the bars indicate significant differences (*p* < 0.01)

To investigate the physiological differences in pear fruits under different canopy architectures, phenotypic parameters were collected at three time points depending on the status of fruit development, i.e., 60 DAF (early development stage), 90 DAF (middle development stage), and 120 DAF (initial ripening stage). Fruit diameter and length increased markedly throughout the entire monitoring period (Fig. 1A and B). Initially, no significant differences were observed between the DP and SP fruits. However, DP fruits subsequently had significantly greater physical parameters than did the SP controls. These data suggest that open-canopy characteristics can effectively promote pear fruit enlargement during fruit development.

The SSC levels increased steadily with increasing fruit development but was only significantly elevated at 120 DAF (Fig. 1C). DP fruits also had significantly greater SSC levels at this stage than did SP fruits. Nevertheless, the amount of TA declined continuously and progressively from 60 DAF until the initial ripening stage; significantly greater levels were observed in DP fruits at the later stages (90 DAF and 120 DAF) than in the control fruits (Fig. 1D). Apparently, the DP canopy environment can transiently increase the SSC level at the initial ripening stage and stably inhibit TA reduction during the later stages of fruit development.

The physiological parameters were not significantly different between IN and EX at most time points, with a few minor exceptions: the values of fruit diameter (SP fruits) and the levels of SSC were greater in EX than in IN at 120 DAF (Fig. 1A and C).

### Analysis of differentially expressed metabolites in pear fruits under different canopy architectures

To identify the macromolecules related to changes in fruit quality in response to the canopy environment, we performed metabolomic analysis via LC–MS between DP and SP fruits at 120 DAF. A total of 5719 and 5065 ions (LC–MS peaks) were detected in positive and negative ionization modes, respectively, across all samples. Among these metabolites, 420 and 451 were annotated in positive and negative ionization mode, respectively. The orthogonal partial least squares-discriminant analysis (OPLS-DA) model from both ion modes clearly distinguished DP fruits from control (SP) fruits (Additional file 2a/c/e/g). The goodness of fit of these models was cross-validated by permutation tests (n = 200), which showed that the predicted variance (R^2^Y values) and predictive ability (Q^2^ values) of the original models were better than those of the permutated models and indicated good predictive ability (Additional file 2b/d/f/h). The initial work demonstrated that the metabolic profiles acquired by LC–MS contain underlying bioinformation that can distinguish between the DP and SP groups.

**Fig. 2:**
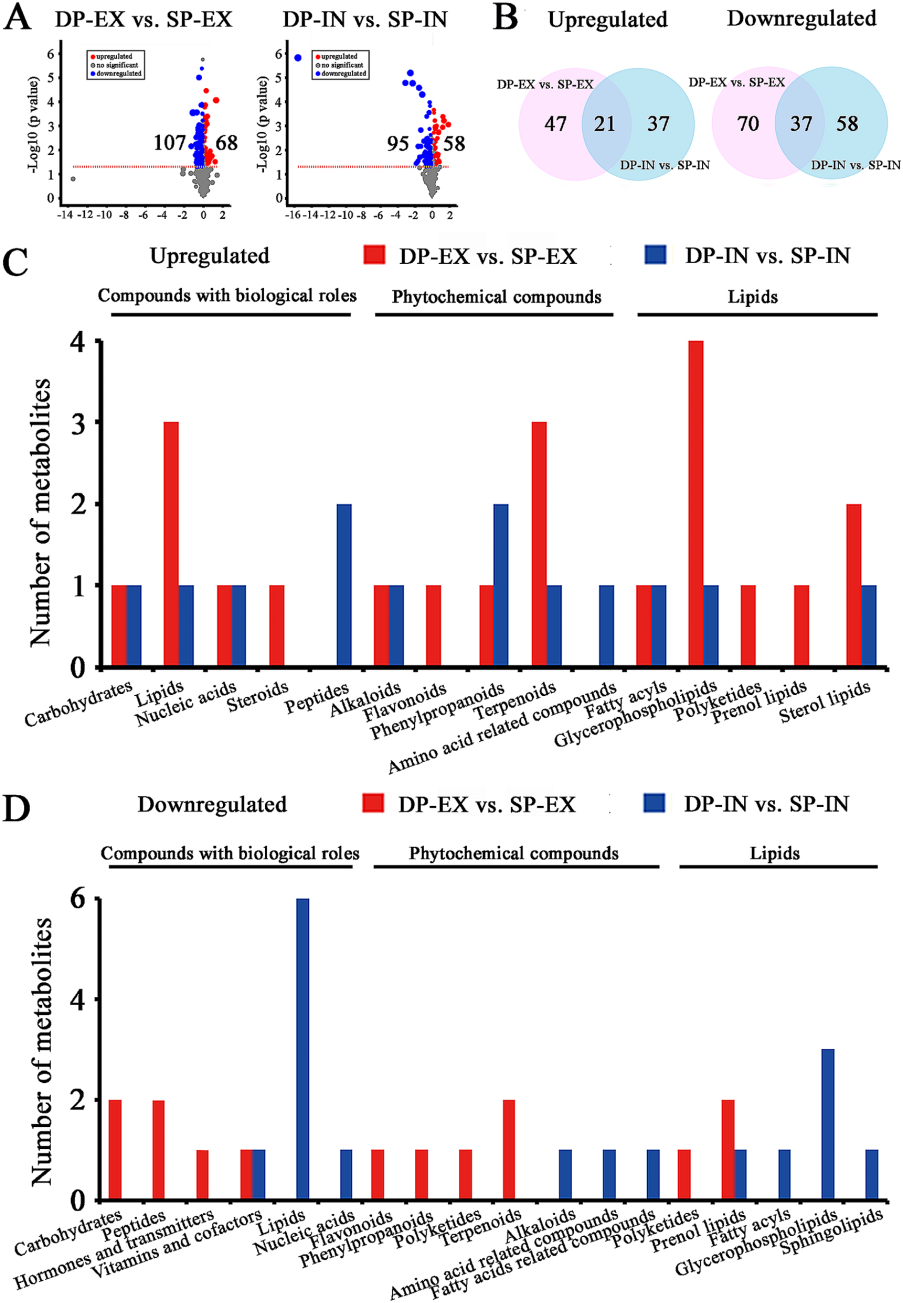
Comprehensive metabolomic profiles differentiating DP fruits from SP fruits. (**A**) Volcano plots indicating the number of metabolite features that were significantly altered between DP-EX/IN and SP-EX/IN. (**B**) Numbers of differentially expressed metabolites. A Venn diagram was constructed to display the overlap between these comparison groups. Numbers of upregulated (**C**) and downregulated (**D**) metabolites at the class level. The names above the horizontal lines represent the class. DP/SP-EX: exterior part of the canopy for DP (flat-type trellis system)/SP (traditional freestanding system); DP/SP-IN: interior part of the canopy for DP/SP

Considering only the identified metabolites, 68 upregulated and 107 downregulated metabolites were detected between the DP-EX and SP-EX fruits (Fig. 2A). In the case of the DP-IN vs. SP-IN comparison, the contents of 58 and 95 metabolites were significantly elevated and decreased, respectively. Venn diagram analysis revealed an overlap of 21 upregulated and 37 downregulated metabolites between DP-EX and SP-EX and between DP-IN and SP-IN (Fig. 2B). The DP-EX fruits displayed upregulated levels of carbohydrates (trehalose), nucleic acids (cytidine), alkaloids (oripavine), glycerophospholipids (lysophosphatidylcholine 15:0), and sterol lipids (cycloartenol) and downregulated levels of prenol lipids (deinoxanthin) (Fig. 2C and D). These significantly differentially expressed metabolites were consistent with the identified differentially abundant metabolites between DP-IN fruits and the controls, indicating that a DP system with open-canopy characteristics may promote the accumulation of certain nutritional components, which might be responsible for the improvement in the quality of the pear fruits.

**Fig. 3:**
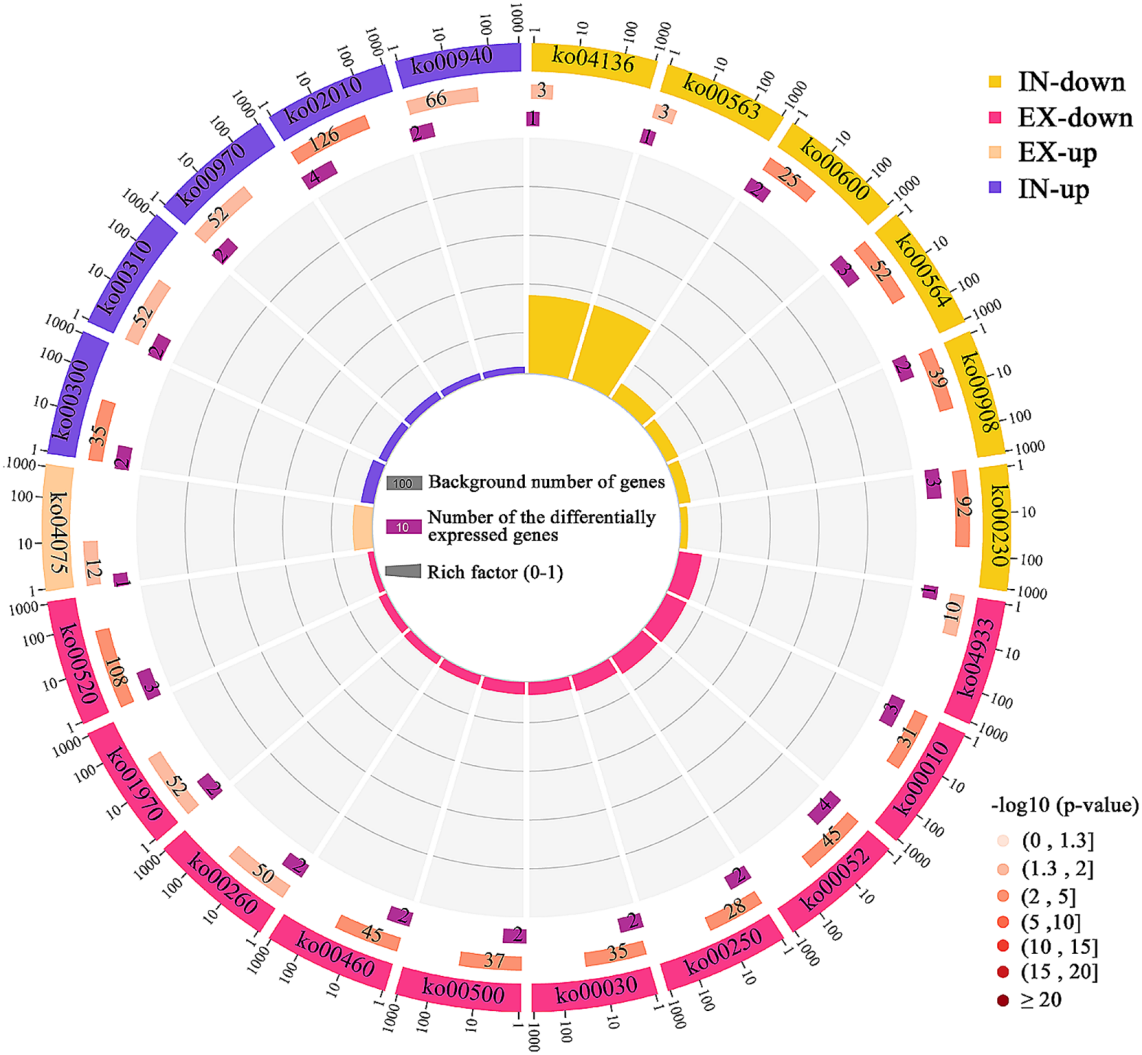
Pathway enrichment analyses of differentially regulated metabolites. The outermost circle shows the classification of the four datasets. Light salmon (EX-up) and deep pink (EX-down) represent upregulated and downregulated metabolites, respectively, between DP-EX and SP-EX. Blue-violet (IN-up) and yellow (IN-down) represent upregulated and downregulated metabolite datasets, respectively, between DP-IN and SP-IN. DP/SP-EX: exterior part of the canopy for DP (flat-type trellis system)/SP (traditional freestanding system); DP/SP-IN: interior part of the canopy for DP/SP. The second circle indicates the background number of metabolites mapped to a certain pathway and *P* values for enrichment of the differentially expressed metabolites for the specified pathway. With respect to the colour gradient from dark to light, the smaller the value is, the darker the colour. The third circle shows the number of differentially regulated metabolites mapped to a certain pathway. The fourth circle indicates the enrichment factor value of each pathway. The enrichment factor indicates the ratio of the number of differentially regulated metabolites mapped to a certain pathway to the background number of metabolites mapped to that pathway

Subsequent KEGG analysis revealed that 22 pathways concerning the nutritional composition of the pear fruits were significantly enriched (Fig. 3). The upregulated metabolites between DP-treated fruits and control fruits were involved in pathways related to ‘plant hormone signal transduction’ (ko04075), ‘lysine biosynthesis’ (ko00300), ‘phenylpropanoid biosynthesis’ (ko00940), ‘lysine degradation’ (ko00310), ‘aminoacyl-tRNA biosynthesis’ (ko00970) and ‘ABC transporters’ (ko02010). Moreover, the downregulated metabolites involved in 16 pathways included ‘glycolysis/gluconeogenesis’ (ko00010), ‘starch and sucrose metabolism’ (ko00500), ‘amino sugar and nucleotide sugar metabolism’ (ko00520), ‘alanine, aspartate and glutamate metabolism’ (ko00250), ‘glycine, serine and threonine metabolism’ (ko00260), ‘galactose metabolism’ (ko00052), ‘glycerophospholipid metabolism’ (ko00564) and ‘purine metabolism’ (ko00230). The overrepresented pathways reflected the regulatory effects of the canopy environment on amino acids, carbohydrates and energy.

### Transcriptomic analysis of pear fruits under distinct canopy structures

To obtain more comprehensive profiles of pear fruits under distinct canopy structures, we also performed RNA-seq analysis comparing gene expression changes between DP and SP fruits at two fruit developmental stages, 90 DAF and 120 DAF. After sequencing the cDNA libraries, the number of clean reads per library ranged from 42.62 million to 51.01 million, and at least 98.04% of the reads in each library were assigned a Q20, indicating high-quality sequencing (Additional file 3). We found that 64.11–76.25% of the clean reads in the libraries were mapped onto the pear reference genome. Based on the FPKM values, we determined the number of genes expressed (FPKM > 1) in individual fruit samples (Additional file 4 A). In total, 25,611 and 25,180 genes were found to be expressed in SP fruits at 90 DAF and 120 DAF, respectively. Similarly, 22,397 and 23,541 genes were identified in the samples from the respective stages of DP fruits. We also observed similar distributions of gene expression levels across all the samples. Approximately 62.5% of the expressed genes were in the 1–10 FPKM range, and 32.3% of the expressed genes were in the 10–100 FPKM range. PCA revealed clear differences between the SP and DP groups as well as between the two fruit developmental stages (Additional file 4B).

**Fig. 4:**
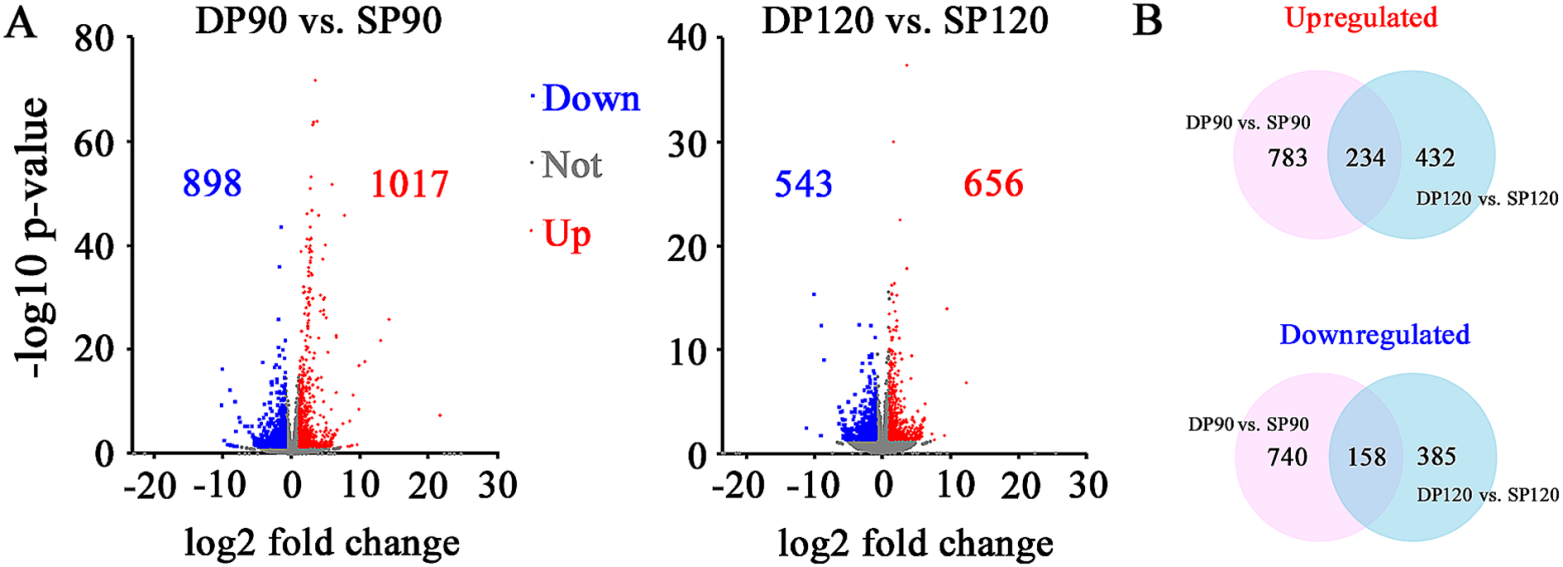
Identification of genes differentially expressed between DP and SP fruits. (**A**) Volcano plots showing genes differentially expressed between DP and SP fruits. The red and blue dots represent the up- and downregulated genes, respectively. (**B**) Venn diagram showing the number of genes with differential expression between the SP and DP systems at the two fruit developmental stages

A total of 1915 (with 1017 upregulated and 898 downregulated) and 1199 (with 656 upregulated and 543 downregulated) differentially expressed genes (DEGs) were identified at the 90 DAF and 120 DAF stages, respectively (Fig. 4A). Among these DEGs, 234 and 158 genes were commonly upregulated and downregulated, respectively, at the two developmental stages (Fig. 4B). These DEGs were considered key regulators in response to the canopy environment. To confirm the reliability of the RNA-seq data, we further selected nine interesting DEGs (four upregulated and five downregulated) to validate the sequencing results (Additional file 4C). All of these genes are known to be related to cell wall organization and modification (*PpEXP*, expansin protein, LOC103964991 and LOC103951053; *PpPEI*, pectinesterase inhibitor LOC103943418), carbohydrate metabolic process (*PpG3PDH*, glycerol-3-phosphate dehydrogenase, LOC103927132), response to auxin (*PpARG7*, indole-3-acetic acid-induced protein, LOC103938998 and LOC103957861; *PpSAUR*, auxin-induced protein, auxin-induced protein, LOC103957879), and photosynthesis (*PpPsbR*, 10-kDa Photosystem II polypeptide, LOC103951972; *PpTHF1*, protein thylakoid formation, LOC103942051). Consistently, the results of the qRT–PCR assay exhibited the same trend and were consistent with the RNA–seq data, validating the reliability of the RNA–seq data.

**Fig. 5:**
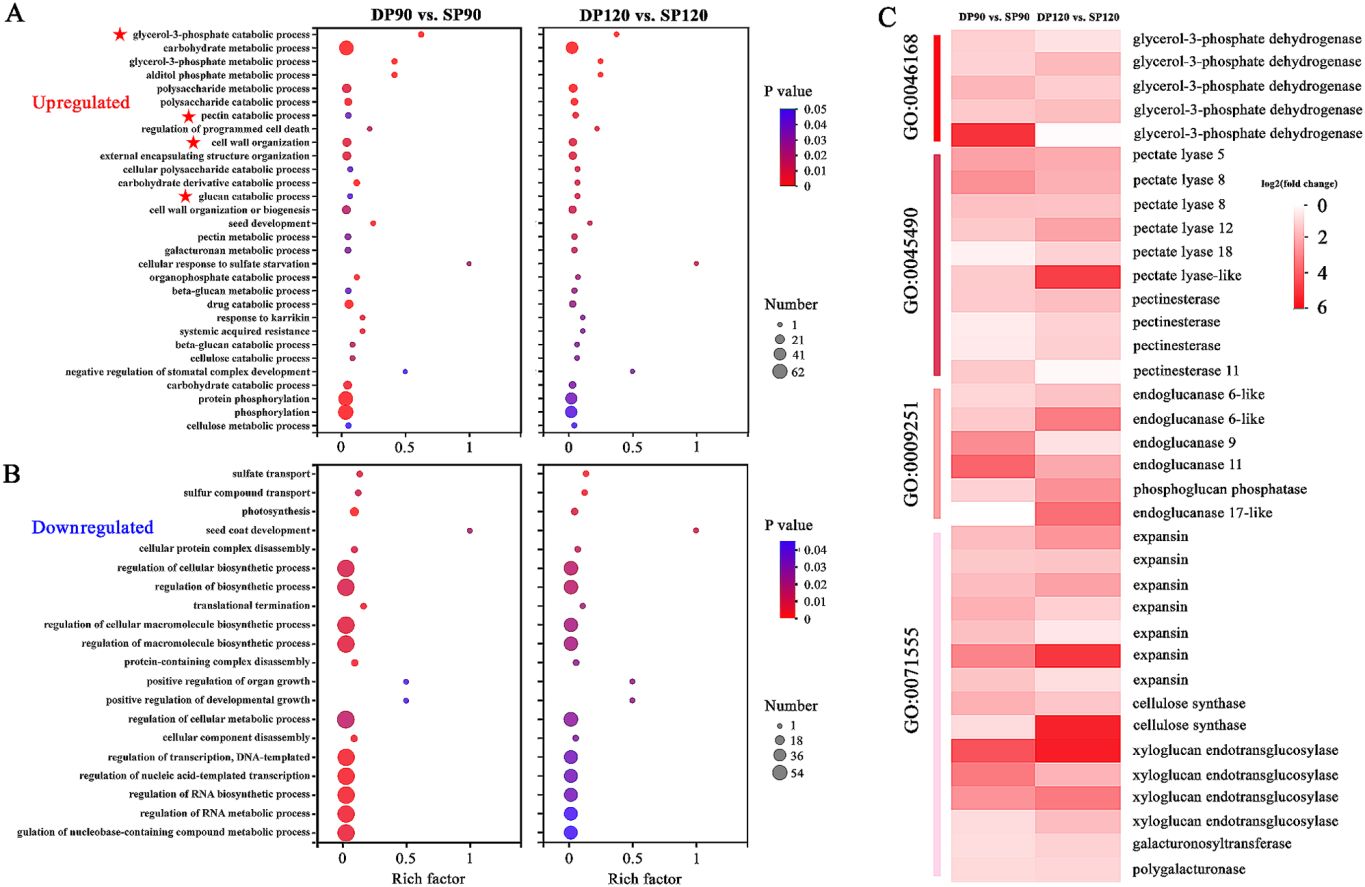
Gene Ontology (GO) enrichment analysis of upregulated (**A**) and downregulated genes (**B**) between the traditional freestanding system (SP) and the flat-type trellis system (DP) at 90 DAF (days after flowering) and 120 DAF. The common GO terms were identified between the two fruit developmental stages. The bubble colour and size correspond to the *p* value and the number of genes enriched in the term, respectively. The richness factor indicates the ratio of the number of differentially expressed genes annotated in a certain term to the number of background genes annotated in that term. Red stars indicate GO terms of special interest. (**C**) Heatmap showing the expression of upregulated genes, which contributed to the enrichment of these GO terms of special interest, at two fruit developmental stages. The red-to-white scale represents a decreasing log_2_-fold change in gene expression in the DP system compared with the SP system

To gain insight into the biological significance of the DEGs, GO enrichment analysis was carried out to determine important functional categories. A total of 30 biological processes were commonly enriched among the upregulated DEGs in the DP fruits at both the 90 DAF and 120 DAF stages (Fig. 5A). GO enrichment analysis suggested that the upregulated genes were associated with organic substance catabolic processes, including ‘glycerol-3-phosphate catabolic process’ (GO:0046168), ‘pectin catabolic process’ (GO:0045490), ‘glucan catabolic process’ (GO:0009251) and ‘cell wall organization’ (GO:0071555). Five *PpG3PDHs* (LOC103959697, LOC103960957, LOC103927132, LOC103953210 and LOC103932939) encoding glycerol-3-phosphate dehydrogenase; six *PpPELs* (LOC103949854, LOC103952213, LOC103959417, LOC103965700, LOC103932918 and LOC103958414) encoding pectate lyase; four *PpPME1s* (LOC103943418, LOC103967214, LOC103948035 and LOC103928018) encoding pectinesterase; five *PpEGs* (LOC103949220, LOC103954973, LOC103956569, LOC103938177 and LOC103928403) encoding endoglucanase; and seven *PpEXPs* (LOC103951053, LOC103954300, LOC103964991, LOC103935534, LOC103940853, LOC103944903 and LOC103948431) encoding expansin proteins all contributed to the enrichment of these GO terms (Fig. 5C). On the other hand, 20 biological processes, including ‘sulfur compound transport’ (GO:0072348) and ‘photosynthesis’ (GO:0015979), were significantly overrepresented among the downregulated genes at both the 90 DAF and 120 DAF stages (Fig. 5B). The overrepresented biological processes emphasize the importance of carbohydrates in pear quality and reveal potentially important genes that may control fruit enlargement.

**Fig. 6:**
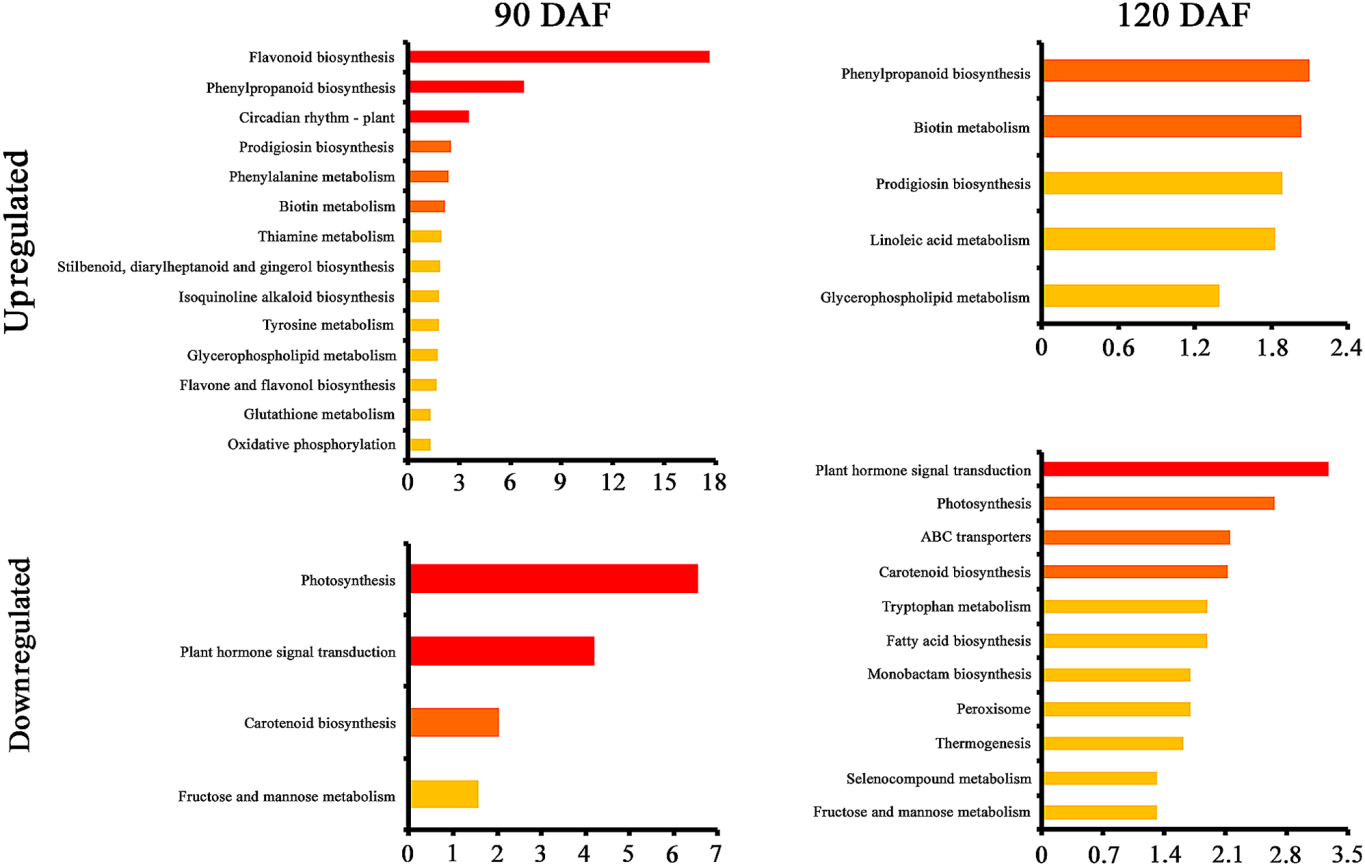
KEGG enrichment analysis of the genes differentially expressed between SP (traditional freestanding system) and DP (flat-type trellis system) at 90 DAF (days after flowering) and 120 DAF. The x-axis is the –log_10_ (*p* value). The colour gradient from red to yellow indicates decreasing significance levels, i.e., red = most significant, orange = moderate, and yellow = least significant

Pathway enrichment analysis revealed that eight perturbed pathways (four upregulated and four downregulated) had lower p values between DP and SP fruits at both developmental stages (Fig. 6). The enriched pathways included ‘phenylpropanoid biosynthesis’ (ko00940), ‘glycerophospholipid metabolism’ (ko00564), ‘plant hormone signal transduction’ (ko04075), and ‘fructose and mannose metabolism’ (ko00051). Compared to those of the controls, the DPs presented several significantly altered mRNAs related to auxin signal transduction, including downregulated *PpAux/PpIAAs* (LOC103929778, LOC103957324 and LOC103954683), *PpGH3* (LOC103965914 and LOC103947840) and *PpSAUR* (LOC103967982, LOC103957861 and LOC103957879).

### Combined metabolomic and transcriptomic analysis at the pathway level revealed that the canopy environment regulates the key genes responsible for fruit taste

**Fig. 7:**
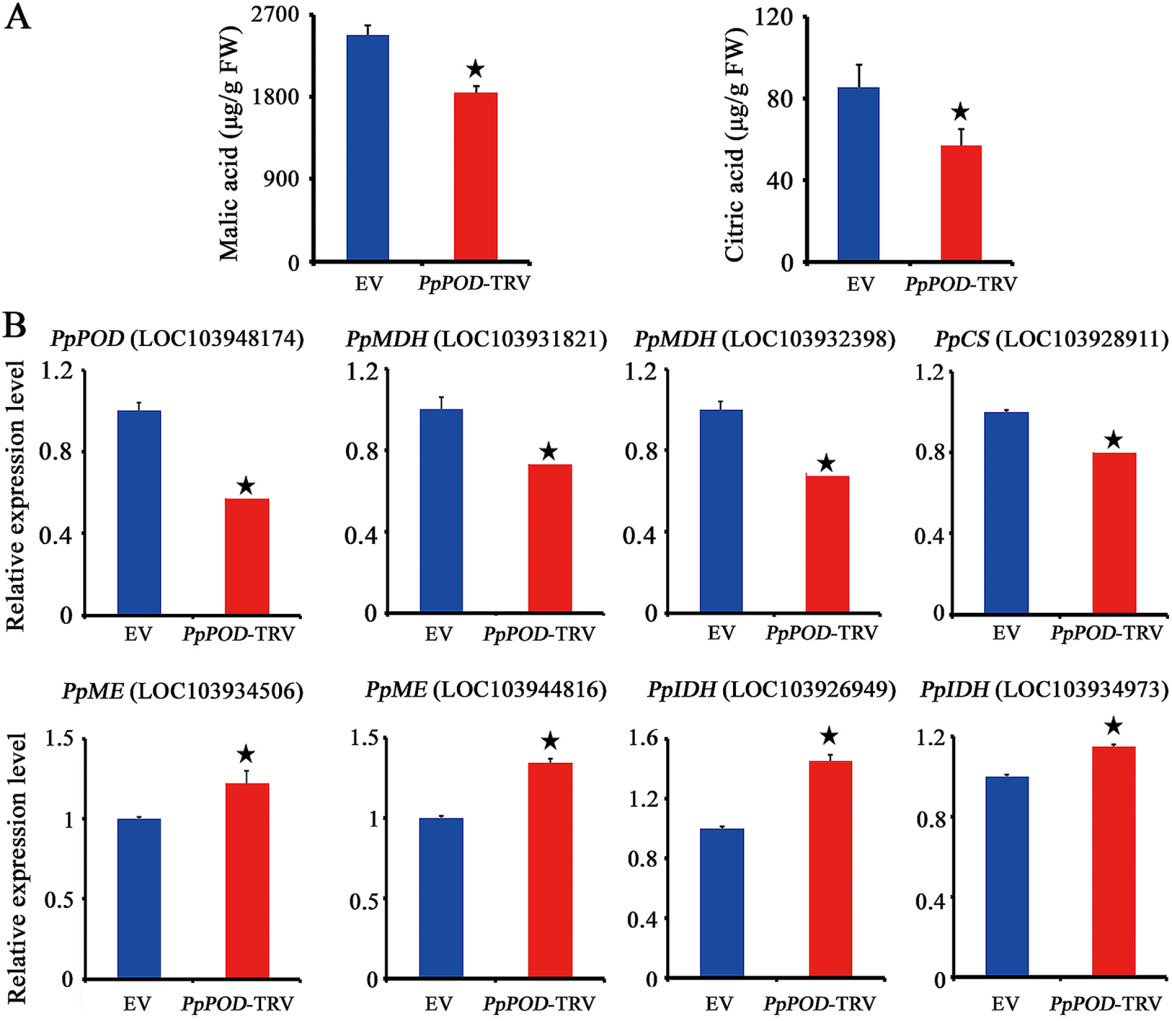
Contents of organic acids (**A**) and expression levels of organic acid metabolism-related genes (**B**) in *PpPOD*-silenced (*PpPOD*-TRV) and control (EV) pear fruits. All the data are from six biological replicates and are expressed as the mean ± standard error (n = 6). Asterisks (*) indicate significant differences according to Student’s t test (*p* < 0.05)

We conducted an integrative analysis of the metabolomic and transcriptomic data at the pathway level to obtain valuable information on pear fruit quality. The results showed that the phenylpropanoid biosynthesis pathway was both isolated and identified among the DEGs and DRMs (Figs. 3 and 6). A total of 24 phenylpropanoid biosynthesis-related genes were upregulated in DP fruits, which included eight *PpPOD* (peroxidase) genes, two *Pp4CL* (4-coumarate:CoA ligase) genes, four *PpBGLU* (beta-glucosidase) genes and two *PpHCT* (shikimate O-hydroxycinnamoyltransferase) genes. We focused on the characteristics consumers desire, such as fruit size and taste. Previous studies showed that *POD* (peroxidase) might play an important role in the accumulation of organic acid [35, 36]. Based on the above findings, we determined that the potential role of *PpPOD* was worth exploring. Of the eight identified *PpPOD* genes, one (LOC103948174) exhibited relatively high transcript abundance levels in all tested fruits. Hence, we constructed a *PpPOD* VIGS vector and evaluated its biological role by using RNA interference (RNAi) on the flesh of pear fruitlets. The results showed that the content of malic acid (2477.93 ± 108.56 µg/g) was significantly greater than that of citric acid (85.34 ± 11.16 µg/g) in the empty control (EV) lines (Fig. 7A). Reduced levels of malic acid and citric acid were observed in fruits inoculated with the *PpPOD*-TRV construct compared with those in the EV lines. The expression of *PpMDH* (encoding malate dehydrogenase, LOC103931821 and LOC103932398) and *PpCS* (encoding citric synthase, LOC103928911), which are implicated in malic acid and citric acid synthesis, was downregulated, whereas the expression of *PpME* (encoding the malic enzyme, LOC103934506 and LOC103944816) and *PpIDH* (encoding isocitrate dehydrogenase, LOC103926949 and LOC103934973), which contribute to their degradation, was upregulated in the *PpPOD*-TRV fruitlets (Fig. 7B). These results indicate that *PpPOD* appears to have a positive regulatory effect on major organic acid accumulation in pear fruit during development and is associated with regulating the expression of organic acid metabolism-related genes.

## Discussion

Training systems manipulate the canopy architecture to maintain optimal levels of light interception and distribution and facilitate high yields of high-quality fruit [20]. To gain insight into the potential mechanisms by which canopy architectures influence pear fruit quality, we performed physiological, metabolomic and transcriptomic analyses of pear fruits grown in distinct training systems.

Fruit size is a key indicator of pear fruit yield and quality. The final size of fruits is determined by both the cell number and the cell size, which are attributed to the processes of cell division and expansion, respectively [37, 38]. Our results demonstrated that the canopy architecture significantly affects fruit size (Fig. 1A and B). GO enrichment analysis revealed significant enrichment of terms associated with fruit ripening, such as “cell wall organization” and “polysaccharide catabolic process” (Fig. 5A). Changes in fruit texture and softening are believed to result from disassembly of the primary cell wall, which is typically accompanied by extensive depolymerization of several classes of cell wall polysaccharides, such as pectins and celluloses [39, 40]. We found that seven *PpEXPs*, which encode expansin proteins, and two *PpCesA* genes, which encode cellulose synthases, were upregulated in DP fruits, which are larger than SP fruits (Additional file 4C and Fig. 5C). Expansins are proteins that mediate cell wall loosening during several phases of plant growth and development [41, 42]. The expression of *EXP* genes during fruit development suggests a role in fruit expansion and ripening in several fruit crops. For example, the gene expression of two expansin genes, *Pa-Exp1* and *Pa-Exp2*, was positively correlated with fruit size during the ripening process in apricot [43]. A larger fruit size was also found to be related to higher transcript levels of *EXP* genes in citrus [44]. In transgenic tomato plants, the suppression of *SlExp1* is correlated with increased fruit firmness and overexpression with enhanced softening, indicating a role for *SlExp1* in fruit ripening [45]. Since expansins can enhance cell wall extensibility and induce cell expansion, we hypothesize that the larger fruit size of DP pear is related to the greater accumulation of *PpEXP* genes.

In addition, fruit development may also rely heavily on the regulation of plant hormones [46, 47]. The ‘plant hormone signal transduction’ pathway was found to be enriched, possibly due to changes in endogenous hormone levels under distinct training systems (Fig. 6). The expression of genes involved in auxin signalling was downregulated in DP fruits (Additional file 4 C), including three *Auxin/Indole-3-Acetic Acid* (*PpAux/PpIAAs*) genes, which are thought to impact the transcriptional activity of target genes by dimerizing with auxin response factor (*ARF*) [48]; two *Gretchen Hagen3* (*PpGH3*) genes involved in the conjugation of free auxin [49]; and three *small auxin-up RNA* (*PpSAUR*) genes known to induce cell elongation and senescence [50–53]. Auxin is a central phytohormone that exerts pleiotropic effects on plant growth and development by regulating cell division, expansion and differentiation [54, 55]. DP system trees are characterized by higher bending angles of lateral branches supported on wire trellises [17, 21]. Branch bending results in a decreased amount of diffusible IAA and controls excessive vegetative growth [56, 57], thus leading to the promotion of reproductive growth and fruit development, which might at least in part explain the larger fruit size in the DP system.

Light was more evenly distributed throughout the DP canopy than within the central leader [17]. This difference was perhaps due to the enhanced spatial distribution of the canopy. The implications of having a DP canopy with a higher net photosynthetic rate are important for pear fruit quality, as this feature suggests greater allocation of photosynthetic products to ripening fruit [9, 17]. In the present study, the levels of TA were significantly greater in abundance in DP fruits than in control fruits (Fig. 1D). Similar trends have been reported in the literature, demonstrating greater organic acid accumulation in fruit crops under higher solar irradiance [58, 59]. It has been shown that light can induce the expression of phenylpropanoid biosynthesis pathway genes [60–62]. Transcriptome and metabolome analyses revealed that the phenylpropanoid biosynthesis pathway was significantly enriched in DP fruits grown with open-canopy characteristics (Figs. 3 and 6). A previous study also revealed that DEGs and DRMs in grapes grown under different canopy types were significantly enriched in the phenylpropanoid biosynthesis pathway [2]. POD, as a key enzyme in the phenylpropanoid pathway, exhibited a positive correlation with organic acids [35, 36]. The present study indicated that the contents of malic acid and citric acid in the *PpPOD*-silenced fruits were relatively lower than those in the EV-treated fruits, which was associated with upregulated transcripts of organic acid degradation-related genes and downregulated transcripts of organic acid synthesis genes (Fig. 7). These results suggest that *PpPOD* may act as a positive regulator of organic acid accumulation. Malic acid and citric acid are considered the major organic acids in pear fruits and have important effects on organoleptic experience [63, 64]. A more even light distribution and greater light use efficiency of the DP canopy appeared to regulate pear fruit flavour with relatively higher organic acid levels, at least in part by activating the expression of *PpPOD*, which increases malic acid and citric acid contents.

## Conclusions

In summary, we performed an integrated physiological, transcriptomic and metabolomic analysis to investigate the effect of canopy architecture on pear fruit quality. The results indicated that the fruit size, SSC and TA content of pear fruits were significantly under the DP system, especially during the later stages of fruit development. The training systems significantly changed the content of carbohydrates, nucleic acids, alkaloids, glycerophospholipids, sterol lipids, and prenol lipids in the pear fruits. Several organic substance catabolic processes were significantly enriched in the fruits of the DP system. Combined with the results of the metabolomics and transcriptomics analyses, we found that the expression of one important candidate gene in the phenylpropanoid biosynthesis pathway, *PpPOD*, was positively correlated with malic acid and citric acid accumulation. The results of this study are helpful for clarifying the molecular mechanism of the canopy microenvironmental response and provide valuable information for developing new strategies for enhancing the nutritional traits of pear fruits.

### Electronic supplementary material

Below is the link to the electronic supplementary material.

**Additional file 1:** List of primers used in this study

**Additional file 2:** OPLS-DA score plots and validation plots of LC‒MS data obtained from pear fruits of SP and DP in positive (a, b, e, f) and negative ion modes (c, d, g, h)

**Additional file 3:** Summary of RNA-seq read statistics

**Additional file 4:** Global view of the RNA-seq expression data at two fruit developmental stages

## Data Availability

All data generated or analyzed during this study are included in this published article and its supplementary information files. The RNA-seq data are available from the NCBI under the accession number PRJNA967128.

## Abbreviations

SP: traditional freestanding system (delayed-open central leader)
DP: flat-type trellis system (Double Primary Branches Along the Row)
IN: interior part of the canopy
EX: exterior part of the canopy
DAF: days after flowering
VIGS: virus-induced gene silencing
SSC: soluble solid content
TA: titratable acidity
LC-MS: liquid chromatography–mass spectrometry
OPLS-DA: orthogonal partial least squares-discriminant
DRMs: differentially regulated metabolites
KEGG: Kyoto Encyclopedia of Genes and Genomes
PCA: Principal component analysis
FPKM: fragments per kilobase per million reads
DEGs: differentially expressed genes
GO: Gene Ontology
