# Supplementary material for: Comparative physiological, metabolomic and transcriptomic analyses reveal the mechanisms of differences in pear fruit quality between distinct training systems
Source: BMC Plant Biol. 2024 Jan 4;24:28. doi: 10.1186/s12870-023-04716-8 (PMC10765702; doi:10.1186/s12870-023-04716-8)
Supplement: Supplementary file 1 — Additional file 1: List of primers used in this study [file 12870_2023_4716_MOESM1_ESM.docx]

Additional file 1: List of primers used in this study

| Ultility | Primers | Sequence (5'-3') | |
| --- | --- | --- | --- |
|  |  | Forward | Reverse |
| Expression analysis | LOC103964991 | TCTCAGCCTGTCTTCC | CCTTATGCCTCCTCTT |
|  | LOC103951053 | TCTCAGCCTGTCTTCC | CCTTATGCCTCCTCTT |
|  | LOC103943418 | TTTCCGAGGTGGTTAGG | ATCCGCCTGTATCTGC |
|  | LOC103927132 | AGCGTTGAGGGATGAA | AAAGGCTGTCCGAGAA |
|  | LOC103938998 | GAAGAAGAATTTGGGTATG | AAGCGAGAAGTGAGATGA |
|  | LOC103957861 | AAAACAGACGGCTTCA | CACAAACCGCTTCTTC |
|  | LOC103957879 | AGTGAGGCTGAAGAGG | GCAGGGAATTGTTAGA |
|  | LOC103951972 | TTCAAGGTGCAAGCAA | CCATCCCTCAAGTCCAT |
|  | LOC103942051 | AAATCGGTCGTCTCGT | ATGGCTGGAAATGCTC |
|  | LOC103931821 | CGTCGCAGTATCCTGATGTC | GCAGCACCACGTTGTTGA |
|  | LOC103932398 | ACTCTACAGTCCCGATTGCG | CCTCTTCTCGTCGTACTTCCC |
|  | LOC103928911 | CAGGGTTATGGTGTCGGTG | GTAACTGGGAAGGCTACGCT |
|  | LOC103934506 | CTGCTTGCTTGCCTGTAACC | CCCTCTTTTGCCTCAGCC |
|  | LOC103944816 | CTGCTTGCTTGCCTGTAACC | TCCTGCCCAGTAGCCCTCT |
|  | LOC103926949 | GTGAAGCGACCGATGACAG | CTCTGGTCTCATCAGGGGTT |
|  | LOC103934973 | GGGTTGATAGGTGGTTTGGG | AGGGCAGTTGGGTTGGC |
|  | *PbPOD* | GGTTGCCCTTCTAGGAGCT | TTGGGGTCGGGGATTG |
| RNAi transient expression assay | pTRV2-*PbPOD* | GCCTCCATGGGGATCCATACCTTCCTGACCACAATGAGAG | ATGCCCGGGCCTCGAGCTAGTCCCGGATTTTATTGGCAACAT |
