# Supplementary material for: Comparative physiological, metabolomic and transcriptomic analyses reveal the mechanisms of differences in pear fruit quality between distinct training systems
Source: BMC Plant Biol. 2024 Jan 4;24:28. doi: 10.1186/s12870-023-04716-8 (PMC10765702; doi:10.1186/s12870-023-04716-8)
Supplement: Supplementary file 2 — Additional file 2: OPLS-DA score plots and validation plots of LC‒MS data obtained from pear fruits of SP and DP in positive (a, b, e, f) and negative ion modes (c, d, g, h) [file 12870_2023_4716_MOESM2_ESM.docx]

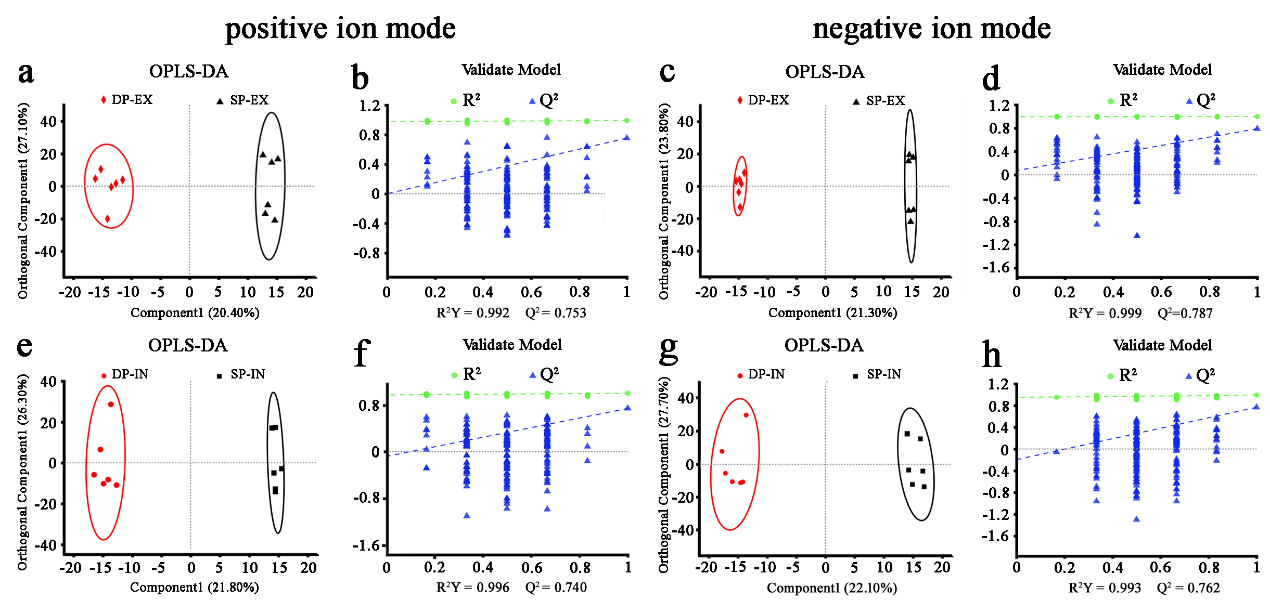


**Additional file** **2. OPLS-DA score plots and validation plots of LC‒MS data obtained from pear fruits of SP and DP in positive (a, b, e, f) and negative ion modes (c, d, g, h).** The metabolic profiles of the DP-EX (red rhombuses), SP-EX (black triangles), DP-IN (red dots), and SP-IN (black squares) groups showed clear differences between the two groups. The ellipses represent the Hotelling T^2^ test with a 95% confidence interval. The OPLS-DA models were cross-validated to evaluate their robustness via a random permutation test (200 cycles). OPLS-DA: orthogonal partial least squares-discriminant analysis, SP: traditional freestanding system, DP: flat-type trellis system, DP/SP-EX: exterior part of the canopy for DP/SP, DP/SP-IN: interior part of the canopy for DP/SP.
