# Supplementary material for: Comparative physiological, metabolomic and transcriptomic analyses reveal the mechanisms of differences in pear fruit quality between distinct training systems
Source: BMC Plant Biol. 2024 Jan 4;24:28. doi: 10.1186/s12870-023-04716-8 (PMC10765702; doi:10.1186/s12870-023-04716-8)
Supplement: Supplementary file 3 — Additional file 3: Summary of RNA-seq read statistics [file 12870_2023_4716_MOESM3_ESM.docx]

Additional file 3. Summary of RNA-seq read statistics.

| Sample ID | Clean reads | Q20 (%) | Total mapped | Multiple mapped | Uniquely mapped |
| --- | --- | --- | --- | --- | --- |
| SP90A | 51005854 | 98.31 | 37708830 (73.93%) | 4244472 (8.32%) | 33464358 (65.61%) |
| SP90B | 44626794 | 98.38 | 33074344 (74.11%) | 3715574 (8.33%) | 29358770 (65.79%) |
| SP90C | 48983662 | 98.36 | 37115967 (75.77%) | 3965950 (8.09%) | 33150017 (67.68%) |
| SP120A | 43871648 | 98.53 | 32433472 (73.93%) | 3757167 (8.56%) | 28676305 (65.36%) |
| SP120B | 49279292 | 98.33 | 37576904 (76.25%) | 4012111 (8.14%) | 33564793 (68.11%) |
| SP120C | 43748638 | 98.46 | 32686642 (74.71%) | 3602611 (8.23%) | 29084031 (66.48%) |
| DP90A | 48368986 | 98.40 | 31868958 (65.89%) | 4815036 (9.95%) | 27053922 (55.93%) |
| DP90B | 46935870 | 98.25 | 30706140 (65.42%) | 4515173 (9.62%) | 26190967 (55.80%) |
| DP90C | 44291494 | 98.32 | 28394432 (64.11%) | 4345411 (9.81%) | 24049021 (54.30%) |
| DP120A | 47104504 | 98.18 | 34741339 (73.75%) | 4042773 (8.58%) | 30698566 (65.17%) |
| DP90B | 42629896 | 98.40 | 30295577 (71.07%) | 3654546 (8.57%) | 26641031 (62.49%) |
| DP120C | 49724746 | 98.04 | 35267212 (70.92%) | 4419677 (8.89%) | 30847535 (62.04%) |

Samples from the SP (traditional freestanding system) and DP (flat-type trellis system) were collected at 90 DAF (day after flowering) and 120 DAF. Three biological replicates for each sample (replicate 1: SP90A, SP120A, DP90A and DP120A; replicate 2: SP90B, SP120B, DP90B and DP90B; replicate 3: SP90C, SP120C, DP90C and DP90C) were analyzed using RNA-seq.
