# Supplementary material for: Comparative physiological, metabolomic and transcriptomic analyses reveal the mechanisms of differences in pear fruit quality between distinct training systems
Source: BMC Plant Biol. 2024 Jan 4;24:28. doi: 10.1186/s12870-023-04716-8 (PMC10765702; doi:10.1186/s12870-023-04716-8)
Supplement: Supplementary file 4 — Additional file 4: Global view of the RNA-seq expression data at two fruit developmental stages [file 12870_2023_4716_MOESM4_ESM.docx]

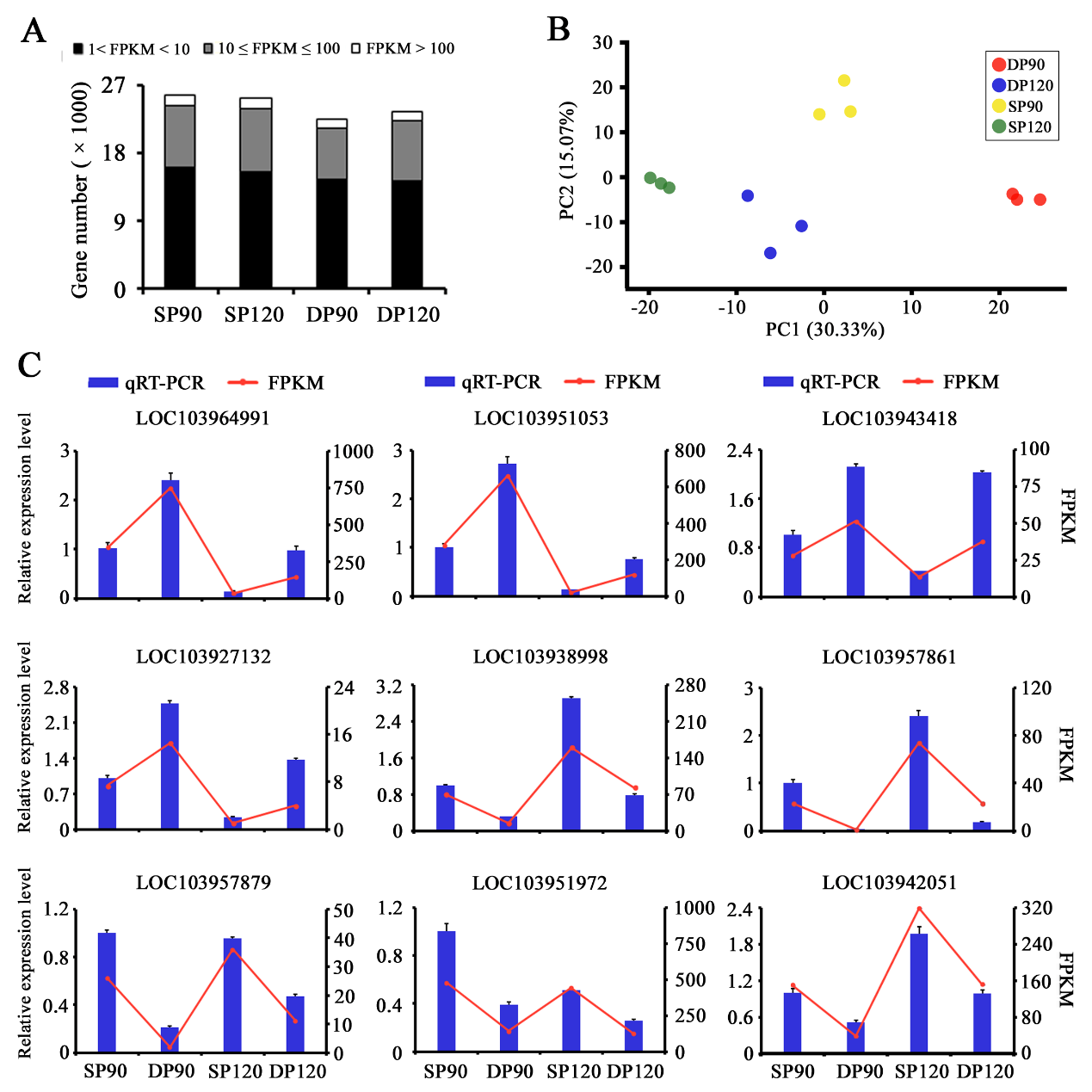


**Additional file 4****. Global view of the RNA-seq expression data at two fruit developmental stages.** (A) Expression levels of genes based on RNA-seq data. Samples from the SP and DP systems were collected at 90 DAF and 120 DAF. SP: traditional freestanding system, DP: flat-type trellis system, FPKM: fragments per kilobase per million reads, DAF: days after flowering. The white, grey and dark segments in each stacked bar indicate the distributions of the different expression levels. (B) Principal component analysis score plot demonstrating the separation of the SP and DP pear fruit samples. Each sample was marked using an individual colour. (C) Verification of the RNA-seq results by qRT‒PCR. Error bars indicate the standard deviation from three biological and technical qRT‒PCR replicates. The Y-axes show the relative gene expression levels determined by qRT‒PCR (blue bars, left) and the corresponding RNA–seq data (red line, right).
